# Supplementary material for: COVID-19 Vaccine Hesitancy and Attitude toward Booster Doses among US Healthcare Workers
Source: Vaccines (Basel). 2021 Nov 19;9(11):1358. doi: 10.3390/vaccines9111358 (PMC8617683; doi:10.3390/vaccines9111358)
Supplement: Supplementary file 1 [file vaccines-09-01358-s001.zip › vaccines-1370839-SI.pdf]

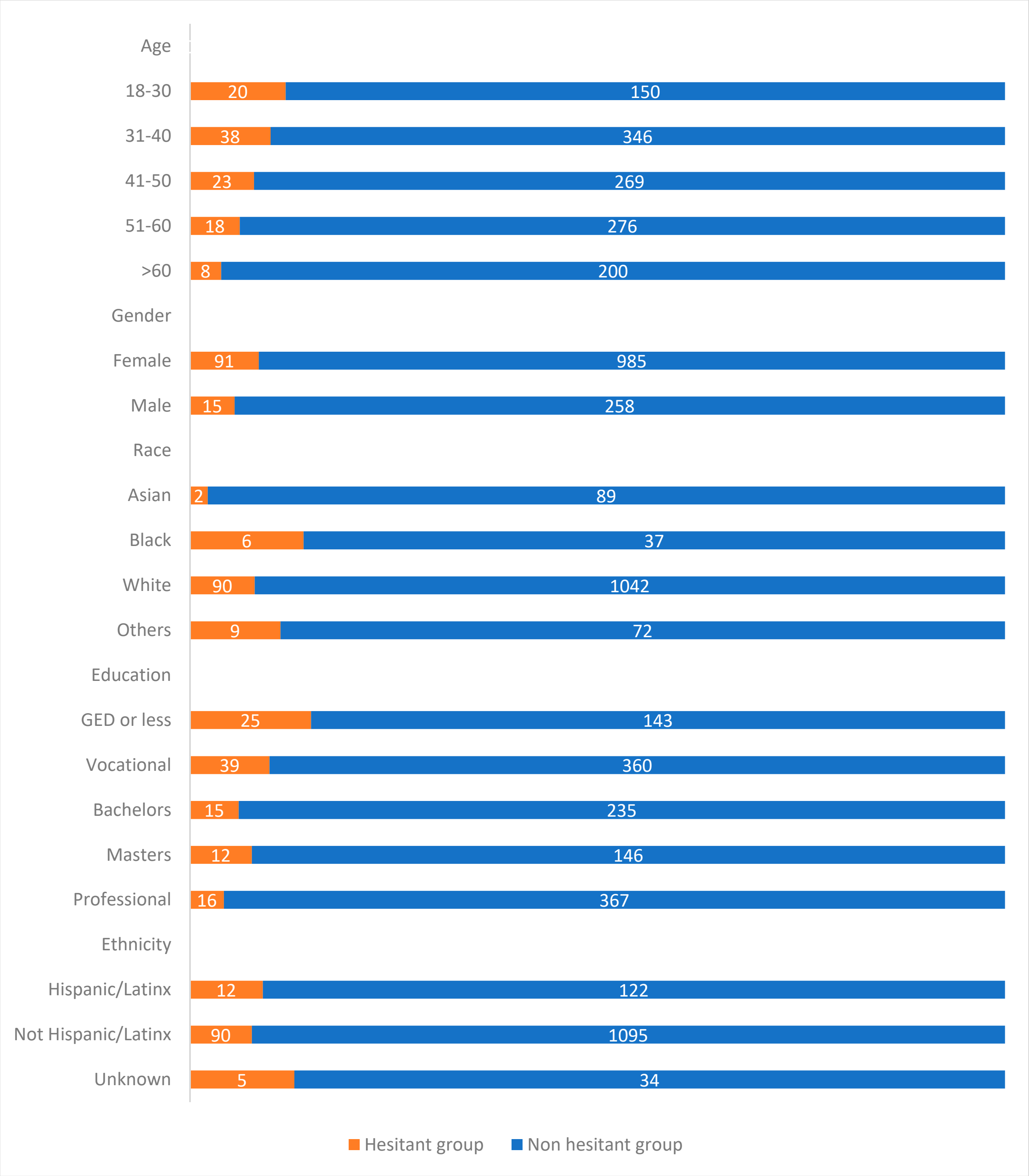

Supplement Figure S1 (A). Proportion of hesitant vs non hesitant group by demographic subgroups.

*While each subgroup varied in absolute number (denoted by white numbers) the figure presents the proportions if each subgroup were equal to allow for better visual comparison,

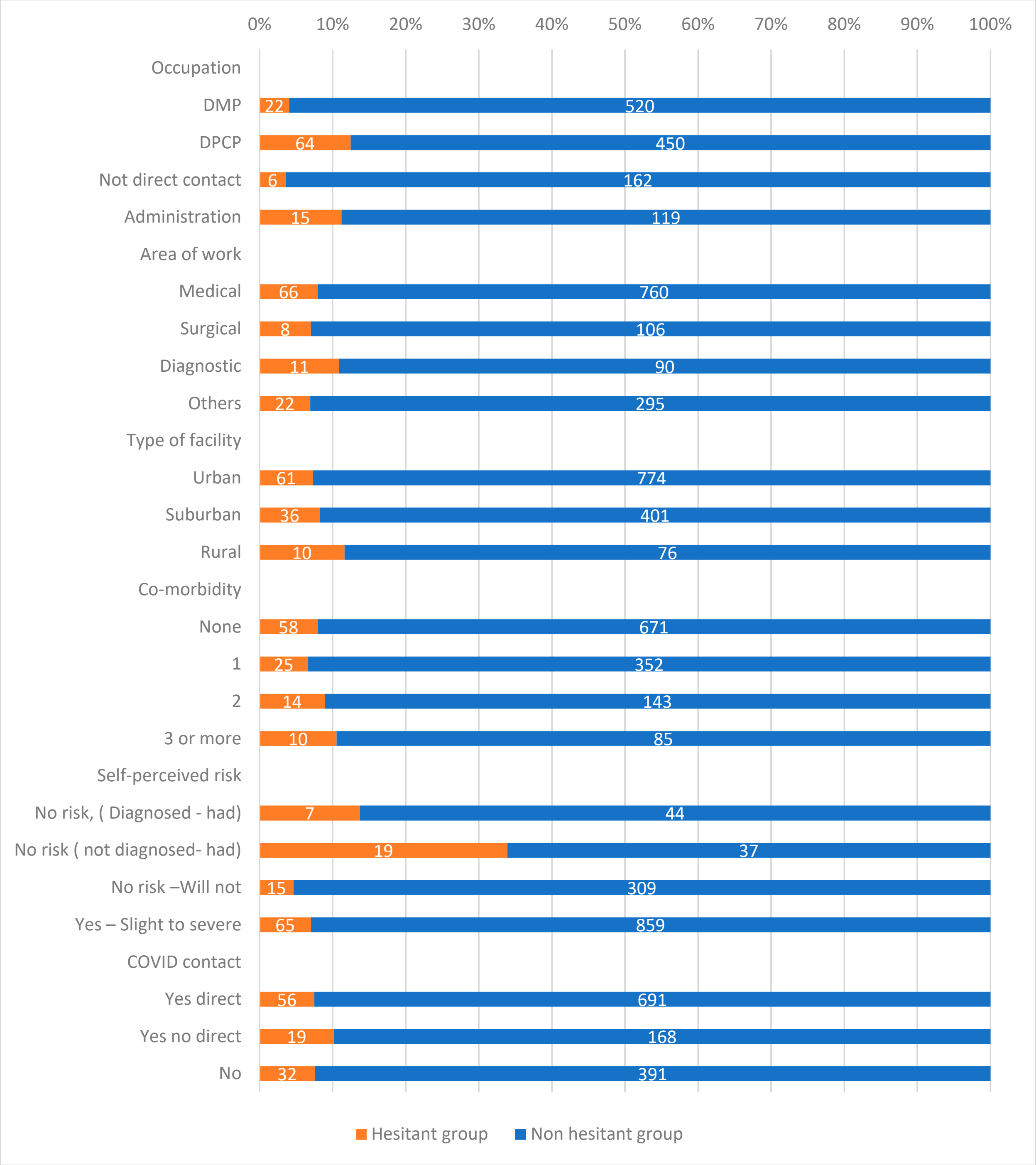

Supplement Figure S1 (B). Proportion of hesitant vs non hesitant group by demographic subgroups.

*While each subgroup varied in absolute number (denoted by white numbers) the figure presents the proportions if each subgroup were equal to allow for better visual comparison,

DMP- Direct medical provider; DPCP – Direct patient care provider
